# What do you do with success? The science of scaling up a health systems strengthening intervention in Ghana

**DOI:** 10.1186/s12913-018-3250-3

**Published:** 2018-06-22

**Authors:** James F. Phillips, John Koku Awoonor-Williams, Ayaga A. Bawah, Belinda Afriyie Nimako, Nicholas S. Kanlisi, Mallory C. Sheff, Patrick O. Asuming, Pearl E. Kyei, Adriana Biney, Elizabeth F. Jackson

**Affiliations:** 1 Mailman School of Public Health, Columbia University, 60 Haven Avenue B-2, New York, NY 10032 USA; 2 Policy Planning Monitoring and Evaluation Division, Ghana Health Service, Private Mailbag, Accra, Ghana; 3 Regional Institute for Population Studies, University of Ghana, Legon, Ghana; 4 Mailman School of Public Health, Columbia University, 60 Haven Avenue, New York, NY 10032 USA; 5 University of Ghana School of Business, Legon, Ghana

**Keywords:** Ghana, Health system strengthening, Scaling up, Health policy, Implementation research, Embedded science, Community-based primary health care, Research utilization, Plausibility trial, Child survival

## Abstract

**Background:**

The completion of an implementation research project typically signals the end of research. In contrast, the Ghana Health Service has embraced a continuous process of evidence-based programming, wherein each research episode is followed by action and a new program of research that monitors and guides the utilization of lessons learned. This paper reviews the objectives and design of the most recent phase in this process, known as a National Program for Strengthening the Implementation of the Community-based Health Planning and Services (CHPS) Initiative in Ghana (CHPS+).

**Methods:**

A mixed method evaluation strategy has been launched involving: i) baseline and endline randomized sample surveys with 247 clusters dispersed in 14 districts of the Northern and Volta Regions to assess the difference in difference effect of stepped wedge differential cluster exposure to CHPS+ activities on childhood survival, ii) a monitoring system to assess the association of changes in service system readiness with CHPS+ interventions, and iii) a program of qualitative systems appraisal to gauge stakeholder perceptions of systems problems, reactions to interventions, and perceptions of change. Integrated survey and monitoring data will permit multi-level longitudinal models of impact; longitudinal QSA data will provide data on the implementation process.

**Discussion:**

A process of exchanges, team interaction, and catalytic financing has accelerated the expansion of community-based primary health care in Ghana’s Upper East Region (UER). Using two Northern and two Volta Region districts, the UER systems learning concept will be transferred to counterpart districts where a program of team-based peer training will be instituted. A mixed method research system will be used to assess the impact of this transfer of innovation in collaboration with national and regional program management. This arrangement will generate embedded science that optimizes prospects that results will contribute to national CHPS reform policies and action.

## Background

Scientists completing work on successful experimental health systems studies often recommend scaling-up results, thereby ending research by handing over lessons learned to policy makers and managers [1]. This paper presents a contrasting paradigm: researchers, policy makers and managers who have completed a successful experiment will now begin a partnership of action and research for developing and testing strategies for guiding the process of scaling it up. This program represents a new phase in a two decade process of implementation science for producing outcomes and actions that have guided the development of community-based primary health care in Ghana [2].

### Community-based health planning and services (CHPS)

Representing Ghana’s flagship approach to achieving Universal Health Coverage, Ghana’s Community-based Health Planning and Services (CHPS) was launched in 2000 as a program for scaling-up community-based primary health care strategies that had been proven to be effective by an experimental study of the Navrongo Community Health and Family Planning Project [3–5]. A series of replication projects launched in response to Navrongo research showed that its strategies represented a replicable approach to basic curative and preventive integrated care that could improve health and reduce childhood mortality and fertility [6–8].

While CHPS has remained a signature achievement of the Ghana Health Service (GHS), Ghana has struggled to bring its approach to primary health services to all who need them, largely because requisite leadership and support systems at the regional, district, and sub-district levels have been neglected. Evidence reported by national monitoring systems in 2008 showed that the pace of CHPS scale-up was progressing so slowly that targeted coverage would require nearly five decades of effort if rates of scale-up at that time were to continue without reform [9]. Moreover, evidence from field research showed that implementation had drifted from the original proven package of implementation strategies [10, 11]: National strategies for scaling up CHPS focused on policy pronouncements, workshops, and didactic leadership training, each pursued as isolated activities that lacked systems perspectives. Policies were grounded in evidence, but strategies for sharing evidence were unlinked to practical demonstration of implementation in the field. University programs, which could bridge leadership gaps by training health specialists, were training health science specialists rather than health systems managers with implementation leadership skills. This resulted in a fundamental disconnect between capacity building, policy making, and evidence-generating field stations.

### The Ghana Essential Health Intervention Program (GEHIP)

In response, the Upper East Regional Health Administration (UERHA) of the Ghana Health Service, in collaboration with the Navrongo Health Research Centre (NHRC) and with technical support from Columbia University’s Mailman School of Public Health, launched the Ghana Essential Health Intervention Program (GEHIP) in 2010 to develop, implement, and evaluate a program of CHPS implementation reform, restructuring, and organizational change [12]. Located in four of Ghana’s most impoverished and remote rural districts, GEHIP implemented a series of health system strengthening initiatives directed at improving leadership and governance systems at all levels of the health system within the district; improving data schemes for informed decision making; designing and implementing emergency referral systems that catalyzed the transport of pregnant women and children to higher levels of the healthcare system where services are more available; and providing catalytic funding that allowed district managers to easily respond to healthcare needs that otherwise would not be addressed with vertical disease-specific allocations.

**Fig. 1:**
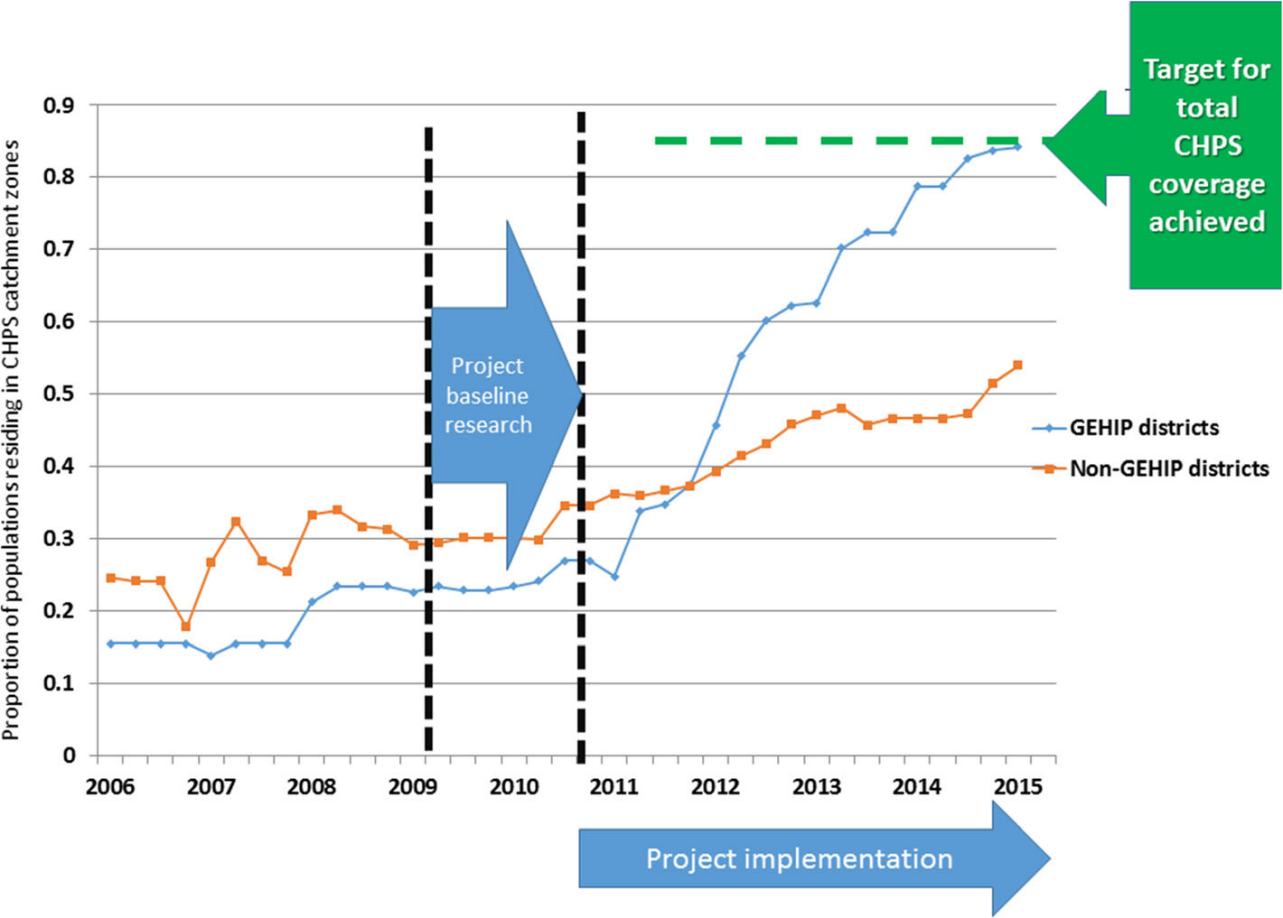
The Percentage of District Populations Covered by Functional CHPS Services in GEHIP Intervention Versus Comparison Districts

After five years of GEHIP implementation, results demonstrated feasible and effective means of accelerating the expansion of CHPS coverage in the intervention districts compared to comparison districts. This expansion resulted in a 49% reduction in under-five mortality in treatment areas relative to levels in comparison districts [13]. GEHIP reduced the time to achieving CHPS-implemented Universal Health Coverage from a national pace that would have required 49 years to a project pace of expansion that achieved this goal in only 5 years *(*Fig. 1*).* Not only was CHPS expanded, but service quality was also enhanced with frontline worker retraining and the addition of emergency public health capabilities [8].

Interventions that enabled these achievements to be attained in five years included strengthening CHPS by expanding the role of community health nurses (including midwives) and volunteers in maternal, newborn and child health, and improving their skills in life saving interventions such as conducting neonatal resuscitation, kangaroo mother care, and care for febrile illnesses. Other interventions included the provision of family planning (FP) and reproductive health (RH) services, training of nurses in Integrated Community Case Management (ICCM), improving the leadership capabilities of nurses and their supervisors through training, and improving logistics supplies and management.

Each participating district was provided with a modest commitment of supplemental funding over a three-year period, providing a basis for exchanges, budgeting, and community engagement to be appropriately focused on CHPS start-up activities. Classroom sessions were minimized; instead, systems strengthening activities were launched in conjunction with community engaged frontline worker training in interventions and peer exchanges to demonstrate teamwork and practical task planning. Emergency referral capacity was instituted with an approach that links worker training to community mobilization and information support; perinatal interventions; and volunteer recruitment, training, and support. The outcome was an approach to integrated services that expanded the coverage and quality of Integrated Management of Childhood Illnesses (IMCI) services and responsiveness to emergency care needs. By linking grassroots politicians to community efforts, the popularity of health development was demonstrated in ways that built political commitment to leveraged financing of health sector investment in CHPS. Community volunteerism, catalytic resources, politically inspired development investment, and sustained diplomatic support from district health officials worked as a system of interaction that transformed CHPS implementation.

### The CHPS+ program

In keeping with Ghana’s legacy of evidence-based health system programming, the GEHIP success is being transitioned into a replication trial phase that will develop, test, and disseminate a strategy for reforming CHPS based on GEHIP lessons learned. Its continuous functioning will provide a learning platform for informing national efforts to scale-up GEHIP strategies through an initiative known as the *Program for Strengthening the Implementation of the Community-based Health Planning and Services Initiative in Ghana (CHPS+).* Launched in 2016 as a five-year project, CHPS+ aims to strengthen the capacity of District Health Management Teams (DHMT) to oversee improvements in the quality of primary health care, focusing in particular on family planning and maternal, newborn and child health care delivery. CHPS+ is comprised of a program of applied learning, team problem solving, peer mentoring, incentivizing financing for improving basic equipment requirements, and technical training that strengthens Ghana’s health system at all levels. It is designed with the intention of decentralizing reform of CHPS implementation activities, with GEHIP lessons learned providing a guide to strategic planning and action. As such, CHPS+ represents a program of research on the utilization of GEHIP research -- the science of which differs from the science of implementing and evaluating a ‘proof of concept’ trial.

This application of implementation research is not new. Organizational change research is a scientific endeavor of management science that is grounded in decades of theory, methods, and application [14–16]. However, scientific investigation of scaling up is only rarely applied to health systems research projects in Africa [17].

### Systems learning districts (SLD)

**Fig. 2:**
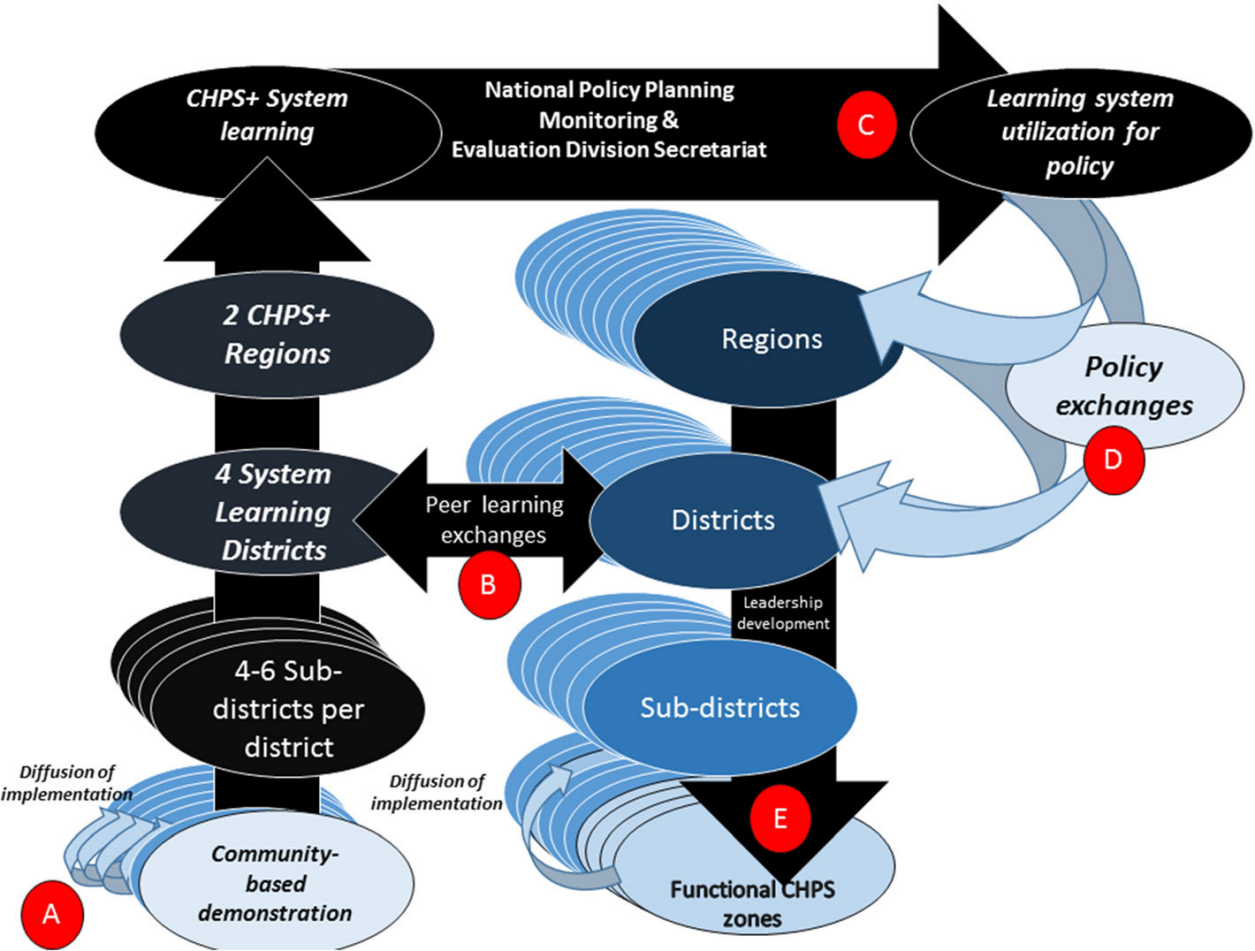
The CHPS+ Theory of Change

CHPS+ is grounded in experience with planned organizational change in Ghana. Taken as a set of activities, Fig. 2 portrays a model for catalyzing implementation-based learning and “guided diffusion” for spreading commitment to community-engaged CHPS implementation and functioning [18–20]. The district management system is the focus of intervention, with district team stakeholders constituting players in the process of instituting large-scale change. To implement the CHPS+ program, key elements of the GEHIP approach will be transferred to regional, district, sub-district, and community-level teams through exchanges that are field based and designed to demonstrate core GEHIP action agenda. Since all district implementers cannot possibly be included in a program of exchange, a program of interchange and outreach is envisioned that will focus on catalytic leadership implementation units that represent the authority and implementation hierarchy in participating districts [21].

Once GEHIP-like implementation is installed in one or two localities of participating districts, the approach will have bridged the implementation knowledge gap: Training will not just convey knowledge and ideas, but will create implementation capacity through demonstration at districts of excellence where operations are fully functioning. Termed *Systems Learning Districts* (SLD) these localities will provide the core capability of the CHPS+ learning system. At each SLD, there will be a program that generates implementation experience with the effective development and functioning of CHPS at the community level (left hand panel, Fig. 2a). Then, with functional CHPS implementation as a learning platform, project resources will be directed to sponsoring exchanges within participating districts so that neighboring communities participate in exchanges via jointly convened public events termed *durbars* where grass-roots political engagement can proceed, local traditional leadership can manifest, and progress with health development can be celebrated by all.

GEHIP has made considerable progress in creating these conditions in four of Ghana’s most impoverished districts. There is a need to convert these districts from experimental zones into SLDs where the elements of system resilience can be demonstrated for replication. The demonstration model will be based on experience gained during the initial years of CHPS workshops, policy instructions, and technical assistance had little lasting impact on the pace of national CHPS introduction. Over the first eight years of operation, 92% of the national CHPS coverage was located in 38 districts that had participated in exchanges organized by the Nkwanta Health Development Centre or the Navrongo Health Research Centre. Systems Learning Districts will be convened to replicate this experience, but apply systems leadership development lessons from GEHIP to the regimen of learning. Rather than imposing a standardized structure for managing CHPS, the SLD will conduct activities that clarify the process of adapting CHPS to local circumstances, capabilities, and needs (Fig. 2b). Implementation development lessons from Nkwanta, will nonetheless be incorporated in the CHPS+ exchange agenda:

i) *Sponsoring “champions of change”*: Invitees will be prioritized according to performance indicators of past commitment to CHPS and ideational leadership in making CHPS work.
ii) *Team building with implementation based learning:* SLD exchanges will focus on building implementation teamwork and avoiding the pitfalls of selecting individuals for workshops that extract individuals from their implementation functions for a few days of didactic training. Participating teams will know in advance that SLD training has resources to commit for implementing a pilot CHPS zone, but activities will proceed with the expectation that teams will observe, learn about, and participate in the progression of implementation milestones that include community, political, and development sector engagement. By teaming participants with SLD implementers, CHPS milestones will be demonstrated via a participatory process rather than through lectures.
iii) *Catalyzing CHPS diffusion:* Trainees will be equipped to develop demonstration and scale-up capacity within their home districts. The goal of each SLD is to ensure that every participating district has a demonstration CHPS zone and capacity to develop at least one demonstration sub-district where implementation processes can be observed for replication. CHPS+ will foster the creation of a system of implementation that extends from doorsteps, to CHPS zones, to sub-district Health Centres, and District Health Management Teams. Systems capacity will set the stage for district-wide CHPS implementation, since pilot capacity can be translated into a participating district program of guided diffusion, whereby communities learn from communities, sub-district teams learn from counterparts, and district managers have an organized resource for accelerating CHPS implementation.
iv) *Improving evidence-based decision-making:* SLDs will have model data systems with capabilities to demonstrate data capture, analysis, and use. All essential primary health care functions will be implemented with technical support from faculty of universities that have public health teaching programs in study regions: The University of Development Studies (UDS) in the Northern Region and the University of Health and Allied Sciences (UHAS) in the Volta Region. All SLDs will have training capabilities, linked to regional training units and counterpart academic units of participating universities. UDS and UHAS are responsible for training the next generation of health professionals in the Northern and Volta regions of Ghana. Columbia University’s Mailman School of Public Health will provide technical support in the areas of implementation, research, and capacity building.
v) *Information for decision-making*. Participants will be oriented to evidence gathering and decision-making, using monitoring tools that have been developed for workers, supervisors, and managers each level of the GHS primary health care system.

The GHS will ensure that SLD-based sub-district and community demonstration zones have a full complement of staffing: community nurses, midwives, supervisors, CHPS coordinators, and fully staff District Health Management Teams. By pursuing these five sets of systems strengthening activities, SLDs will function not only as districts of excellence and innovation for the rest of the districts to emulate, but also as localities where a culture of health service excellence and systems thinking can be demonstrated, studied, and disseminated. The process of CHPS development and reform that was tested by GEHIP, will be converted into a replication strategy that can be used by the national program for accelerating and improving CHPS scale-up (Fig. 2c). To maximize capabilities to replicate GEHIP, teams from each SLD will be taken to the GEHIP intervention districts in the Upper East Region where they will learn about GEHIP development processes from peers who have managed and implemented the program (Fig. 2d). In this manner, CHPS+ will be an experiment in the utilization of an experiment (Fig. 2e).

## Methods/design

### Hypotheses and goals

CHPS+ will test the primary implementation hypothesis that the pace of expansion of CHPS population coverage can be significantly increased relative to the pace of CHPS expansion in districts not yet exposed to CHPS+ interventions. Research is also designed to test the impact hypothesis that CHPS+ implementation will improve infant and child survival. A three component mixed method evaluation strategy has been launched for program evaluation involving: i) baseline and endline randomized cluster sample survey research with 247 census enumeration clusters dispersed in 14 districts of the Northern and Volta Regions for assessing the effect of cluster exposure to CHPS+ activities on childhood survival; ii) a service delivery point monitoring system for assessing the association of changes in service system readiness with CHPS+ interventions; and iii) a program of qualitative systems appraisal (QSA) to gauge stakeholder perceptions of systems problems, reactions to CHPS+ interventions, and perceptions of change. Temporal variance in project implementation will be monitored to provide stepped wedge recorded differential exposure to operations. Integrated survey and M&E data will permit multi-level longitudinal models of impact; longitudinal QSA data will provide data on the implementation process.

The overall goal of the project is to develop sustainable capacity to implement, monitor, and evaluate a health systems strengthening strategy in Ghana that will improve national capabilities to scale-up community-based primary health care coverage, quality, and impact. This approach of phased capacity building for scale-up will create a culture of health service excellence and systems thinking within the GHS and demonstrate the process of community-based primary health care development that has been tested by GEHIP. The project will assemble into a single system of care successful health system strengthening innovations. By integrating capacity-building functions into existing regional and local training institutions, in partnership with a university-based capacity-building program, CHPS+ will pursue the objective of institutionalizing health systems development and build a unified and sustainable Ghanaian system of community-based primary health care. CHPS+ will implement an integrated management approach by bringing together institutions involved in training health professionals, and individuals who have played pioneering roles in health development innovations and strengthening in Ghana.

### Collaborating partners

The implementation of CHPS+ service and systems strengthening activities will be the responsibility of the Ghana Health Service *Policy Planning Monitoring and Evaluation (PPME)* Division*.* This will ensure that CHPS+ builds upon a legacy of implementation expertise, yet coordinates its efforts with the investments and regional programs of other donors and initiatives that must coordinate their priorities and activities with the GHS, enabling the CHPS+ partnership to function more in the manner of a national consortium than a project. The Regional Institute for Population Studies (RIPS) at the University of Ghana will lead the research and evaluation effort for CHPS+ in the two regions.

### Implementation research

#### Quantitative systems monitoring

The Ghana Health Service routinely conducts health management information systems monitoring at all service delivery points. Quarterly reports of service caseload volume by type of services provided are available for all facilities and CHPS zones in the Northern and Volta Regions. This system, known as the “District Health Information Management System” (DHIMS) has been augmented by to simplify and improve the quality of data collected [22, 23]. This simplified system, in turn, has been equipped with geographic positioning system data on all service points [24, 25]. Since DHIMS also registers information on the timing of CHPS implementation milestones, CHPS+ will have the capability of monitoring the pace, content, and coverage of service operations. Using facility survey methods developed by GEHIP, the project has baseline and endline service readiness data linked to DHIMS and available for assessing the impact of project interventions on the quality of care.

#### Qualitative systems appraisal

CHPS+ will conduct longitudinal qualitative research on systems functioning, systems changes, and project processes and implementation impact. Techniques employed, will be adapted from qualitative research tools of the business and organizational research paradigm developed by various authors and applied in various ways as ‘the strategic approach’ [19, 26–30], participatory planning [31, 32], organizational development [33] and people-centered science [34, 35]. In this approach, key stakeholders in the organization are identified and research is applied to gauging reactions, advice, or experience of stakeholders at each level of the system. Since the approach represents an application of open systems theory, community stakeholders are included in the assessment, permitting research outcomes to adapt insights about formal organizational structure and functioning to the social, economic, or political context in which effective functioning can be optimized [14]. In this instance, the application of open systems thinking requires any research on the functioning of CHPS to focus on the social and political system at the community and district levels, not just health system of the GHS. QSA must also be multi-leveled, with qualitative data compiled at the frontline worker, supervisory, and managerial level to structure and define a system narrative on the operational design, functioning and leadership of CHPS.

To monitor and interpret the impact of CHPS+, teams participating in CHPS+ must be the focus of QSA before their exposure to the intervention, immediately following the intervention, and at the end of the project. Examples of this approach, as applied to CHPS have informed the GHS of community and worker perceptions of the appropriate design of operations [36], social constraints to particular strategies [11, 37, 38] worker reactions to CHPS [39], and stakeholder advice and impressions of the impact of interventions on systems change [40].

The baseline phase of the QSA will precede the intervention in order to ensure that procedures for scale-up are tailored to the context. In addition to this, the QSA will also explore community and health care workers’ perceptions about CHPS and CHPS+, and also assess the successes and challenges of the scale up during and after implementation of the interventions.

### Impact research: The household survey

Using questionnaires that are designed to replicate the national 2014 Demographic Health Health Survey, CHPS+ will conduct baseline and endline demographic surveys for assessing impact. The baseline CHPS+ project survey sample is designed to obtain information to detect a 15% reduction in under-five mortality with 80% power at 5% level of significance in each of the two study regions. Overall, the interventions are taking place in eight districts (four in each region) while another eight districts (four in each region) are being used as comparison districts. Survey sampling is designed to draw a representative sample of the number of women of reproductive age (15–49 years) in three of the four treatment districts in each region and in four randomly selected comparison districts. Thus, a total of fourteen districts, seven per region, were included in the baseline survey. A two-stage stratified cluster sampling approach was used. In the first-stage, census enumeration area clusters were sampled, and in the second-stage, households will be sampled from the first-stage clusters. Because a minimum of 30 clusters is conventional for the first stage of cluster sampled household surveys [41], a relatively conservative starting point of 40 clusters each for intervention and control districts in each region was selected.

Because the sample is designed to detect separate under-five mortality effects in each of two regions, sample size calculations were completed independently for each region. Final sample size and sampling parameters were based on region-specific estimates of census enumeration area intra-cluster correlation (ICC) and the number of children under five expected per woman. These parameters were calculated from publicly available data from the 2014 Ghana Demographic and Health Survey [42]. In Volta Region, ICC was higher and the number of children expected per woman was lower than in Northern Region. Therefore, a larger number of clusters and a larger number of households per cluster will be sampled in Volta Region. Sampling requirements for each region were calculated using a software system for conducting power calculations in multi-level randomized experiments known as Optimal Design [43].

#### Sample design

The sampling frame for the first stage was from the 2010 Population and Housing Census which provided a complete listing of Enumeration areas in the fourteen districts to be surveyed. The sampling frame contained information such as the location and estimated number of households. In each region, Enumeration areas were stratified by rural versus urban and by the estimated number of households in the cluster. Clusters were stratified into three groups (small, medium, or large yielding a total of 6 strata in each district. Enumeration areas were sampled from each stratum using probability proportional to population size (PPS). Weights will be applied at the cluster level to standardize probabilities of household selection based on the relative population size of clusters.

To obtain a sampling frame for the second-stage sampling, a household listing of all households in each cluster was compiled based on census information on household size and number women of reproductive age (15–49) in each household. Households were then stratified into three strata defined by the number of eligible women in each household.

To maximize sample efficiency for difference-in-difference estimation at the endline, baseline sample clusters will be reused without modification to permit longitudinal observation of clusters and the assessment of the average treatment effect arising from the timing of exposure of clusters to project interventions. The second-stage sampling procedure will be repeated, yielding a household sample for the longitudinal observation of children exposed versus unexposed to interventions over the duration of the project, with provision for statistical adjustment of baseline and endline differentials and changes in project endpoints that are unrelated to CHPS+ interventions.

#### Assessing impact

CHPS+ will have core, intermediate outcome, and process endpoints, each requiring systems of data capture, data management, and analysis to gauge project impact. These core endpoints and indicators will be consistent with the Ghana Ministry of Health core indicators of health improvement with instruments designed to maximize comparability with national Ghana Demographic and Health Survey instruments. This will involve indicators of under-five mortality, infant mortality, and neonatal mortality by gender of child; age specific and total fertility rates and proximate determinants that are relevant to policy; and indicators of parental health seeking behavior, such as skilled attendant delivery, care of sick children, exposure to community-based services, distance to health facility and utilization of facilities for essential care. Critical covariates essential to the understanding of equity and impact, such as educational attainment, household economic status, and distance to service point will also be assessed.

CHPS+ will utilize impact assessment strategies that have worked well for GEHIP. Routine compilation of time trends indicators will be supplemented with end-of-project *difference-in-differences* calculations for each indicator. This econometric strategy has been applied elsewhere for the assessment of non-randomized plausibility trials [44–46] and successfully applied to the evaluation of GEHIP [47]. The procedures involve collecting data in baseline clusters, monitoring systems changes and the timing of these changes, and repeating the survey in baseline clusters with a separate endline stage two sample to gauge effects. Regression analysis is based on merged baseline and endline data for the estimation of parameters that control for baseline differences and contextual confounders, changes over time that are unrelated to interventions, and conditional effects of interventions that control for these confounding changes by estimating net intervention effects. A difference-in-difference estimate of program effects is given by the child survival effect of CHPS+ systems interventions (**S**) where individual child i is scored 1 if the household is located in a cluster that is exposed to CHPS treatment and zero otherwise and z is scored 1 if the case *i* is observed in the endline and zero if the case is observed in the baseline. If all covariates are equal to zero for child *i*, (*i.e.,* *X*_*1*_*=0*, *X*_*2*_*=0,...* *X*_*k*_*=0,* *S=0,* *Z=0*), the underlying mortality hazard is *h*_0_(t) and the conditional multilevel proportional hazard model is:

1 $$ {\boldsymbol{h}}_{x\sim SZ}\left(\boldsymbol{t}\right)={\boldsymbol{h}}_{\mathbf{0}}\left(\boldsymbol{t}\right){\boldsymbol{e}}^{\left[{\beta}_0+\sum \limits_{k=1}^K{\beta}_k{X}_{ij k}+\gamma {S}_j+\delta {Z}_{ij}+\zeta {S}_j{Z}_{ij}+{\mu}_j+{\varepsilon}_{ij}\right]} $$

for all *t*, where:

*X̃*_*i*__*j*__*k*_ represents *K* characteristics of child *i* in cluster *j*;

*S*_*j*_ indicates whether cluster *j* is in the treatment area (*S* = 1) or comparison area (*S* = 0);

*Z*_*ij*_ indicates whether the person time of child *i* in cluster *j* occurs during the post-treatment period (*Z* = 1) or pre-treatment period (*Z* = 0);

*S*_*j*_*Z*_*ij*_ is a cross-level interaction term of treatment and period for estimating the net effect of treatment in the difference-in-differences approach;

and

*μ*, *ε* represent residuals for cluster and individual levels, respectively.

## Discussion

CHPS+ is an implementation research project that develops and tests a strategy for scaling up community-based primary health care in Ghana. Despite impressive investment of Government of Ghana resources in CHPS program manpower expansion, equipment, and community facilities, the pace of program implementation has been shown in the past to be unacceptably slow. Community-engagement has been neglected, with programs for facility development relying more on contractors than on community commitment to make services work. Manpower for community services is expanding faster than the availability of facilities where workers can be posted. And mounting evidence suggests that the quality of supervision [48] and services [49] is often poor. There is a need to redirect investment into low cost and effective alternative strategies for expanding CHPS operations that have been demonstrated by the GEHIP project in the Upper East Region, but will remain confined to that region unless scaling up strategies are developed and tested. CHPS+ will not only fill an information gap in primary health care development in Ghana, its management will be integrated into national, regional, and district systems of program coordination. As such, it represents an application of embedded science to the process of ensuring essential program ownership of the implementation process [50, 51]. CHPS+ will test a means of accelerating CHPS expansion that is based on GEHIP success, but implemented with an approach that aims to demonstrate large scale action with lifesaving outcomes.

Taken as a system of interventions, training, and program development, CHPS+ will demonstrate a practical approach to evidence-based health systems development in Sub-Saharan Africa. Examples of implementation science for supporting scale-up are urgently needed. Throughout Africa, governments are launching large-scale implementation efforts for achieving “Universal Health Coverage” (UHC) with a priority focus on community-based primary health care as an overarching strategy for implementing this goal [52–54]. Strategic options for this approach vary substantially, with some programs relying upon volunteer community health workers [55], while others advocate the deployment of paid professional paramedics [56]. Training durations, supervisory strategies, compensation levels, and community engagement models can also vary. But, most importantly, approaches that are demonstrated and tested in experimental trials may not be sustainable or replicable at scale. Ghana’s approach to researching the utilization of research, with CHPS+ as its current source of evidence, demonstrates a paradigm for developing a process of programmatic change and development that is grounded in scientific investigation.

## Abbreviations

CHPS: Community-based Health Planning and Services Initiative
CHPS+: Program for Strengthening the Implementation of the Community-based Health Planning and Services Initiative in Ghana
DHMT: District Health Management Teams
FP: Family Planning
GEHIP: Ghana Essential Health Intervention Program
GHS: Ghana Health Service
ICCM: Integrated Community Case Management
IMCI: Integrated Management of Childhood Illness
NHRC: Navrongo Health Research Centre
PPME: Policy Planning Monitoring and Evaluation Division, Ghana Health Service
QSA: Qualitative Systems Appraisal
RH: Reproductive Health
RIPS: Regional Institute for Population Studies
SLD: System Learning District
UDS: University of Development Studies
UHAS: University of Health and Allied Sciences
